# Good to the Last Drop? Nectar Depletion by Insect Pollinators Varies Among Plant Species and Through Time

**DOI:** 10.1002/ece3.74008

**Published:** 2026-07-14

**Authors:** Skylar H. Gillies, Douglas B. Sponsler, Zachary J. Hackworth, Darin J. McNeil

**Affiliations:** ^1^ Department of Forestry and Natural Resources University of Kentucky Lexington Kentucky USA; ^2^ Boys' Latin of Philadelphia Charter School Philadelphia Pennsylvania USA

**Keywords:** bees, butterflies, floral resources, invasive plants, native plants, nectar production

## Abstract

Most insect pollinator species use floral resources exploited by many conspecifics and heterospecifics. In systems where floral resources limit pollinator populations and give rise to competition, understanding whether competition varies across the growing season is a fundamental question in explaining how species co‐exist and how communities are structured. Recent work on nectar depletion rates in plant‐pollinator communities has demonstrated the value of measuring nectar depletion rates as a proxy for competition among pollinators. One hypothesis on pollinator competition, hereafter, “equilibrium hypothesis,” predicts that nectar depletion rates should be constant across the growing season within a given community. In contrast, an alternative hypothesis, hereafter, “dynamic hypothesis” suggests the opposite. To test these hypotheses, we sampled nectar depletion and floral abundance across an early successional plant community over the bulk of a growing season in central Kentucky, USA in spring/summer, 2023. We predicted that a dynamic ecosystem such as that studied here would most likely be characterized by dynamic depletion rates. Initially in the growing season, nectar depletion rates within this early successional vegetation community were moderate (e.g., 50%–70%) but steadily climbed to 85% as the growing season advanced. Plant species varied widely in depletion rate: invasive *Lamium purpureum* being the least depleted (48% depleted) and native *Asclepias syriaca* the most (95% depleted). After correcting for species‐specific abundance, *Vicia sativa* and *Lonicera japonica* produced the greatest volume of nectar over the first 7 weeks, after which time *Asclepias syriaca* and *Monarda fistulosa* yielded a 2–3× increase in available nectar for pollinators. These data add to a growing body of literature suggesting that the dynamic hypothesis explains patterns of competition in early successional plant‐pollinator communities. Importantly, future work should aim to examine nectar depletion dynamics within more stable ecological communities and those with few exotic species.

## Graphical abstract

Understanding how pollinators compete for available floral resources (nectar, pollen, etc.) across time is poorly studied, especially in the western hemisphere. To address this knowledge gap, we measured nectar availability and subsequent depletion as a proxy for resource competition intensity among pollinators in an early successional old field community in central Kentucky, USA. Nectar depletion varied by species and over time in our study, indicating that some floral species may be more sought‐after by pollinators—and thus more valuable in a system.

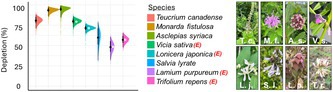

## 1 Introduction

Within temperate plant‐pollinator communities, most insect pollinators compete within and among species for floral resources (Page and Williams 2023; Thomson and Page 2020). While there are many examples of specialist plant/pollinator pairs in ecological communities (e.g., the solitary bee *Andrena erigeniae* [spring beauty miner] and its floral associate *Claytonia virginica* [spring beauty]; Parker et al. 2016), most insect pollinators feed on floral resources that are also used by many other pollinator species (Fontaine et al. 2008; Kaluza et al. 2017). Thus, understanding the extent to which insect pollinators compete and for which resources is central to understanding how pollinating species co‐exist within complex plant‐pollinator communities (Bascompte and Scheffer 2023; Bosch et al. 2009). Regardless, the premise of competition is resource limitation (Roulston and Goodell 2011). In plant‐pollinator systems, resource limitation (and competition) can be most readily observed in the depletion of pollen and, especially, nectar (Sponsler et al. 2023). From an ecological perspective, competition among pollinators can inform the extent to which pollinator communities are regulated by bottom‐up processes (i.e., food availability, as suggested by some; Mitchell et al. 2009; Roulston and Goodell 2011). Or, if not, data on competition can help identify the factors regulating pollinator communities (e.g., disease, availability of nest sites, depredation, etc.; Antoine and Forrest 2021; Goulson et al. 2015). From an applied conservation perspective, understanding competition dynamics is important for a variety of reasons (Steffan‐Dewenter and Tscharntke 2000; Thomson and Page 2020), especially with regards to competition between managed *Apis mellifera* (western honey bee) colonies and wild/native bees (Shavit et al. 2009; Wojcik et al. 2018). Thus, understanding the extent to which floral resources are limiting in pollinator networks is of central interest in both theoretical ecology and applied conservation.

In systems where floral resources limit pollinator populations and give rise to competition, a variety of phenological mechanisms acting on insect populations may yield temporally heterogeneous “demand” for floral resources (CaraDonna et al. 2020; Souza et al. 2018). For example, in eastern North America, a number of authors have described a “summer dearth” phenomenon of resources occurring midsummer between peaks of floral resource availability in the preceding spring or the ensuing fall (Aldridge et al. 2011; Bishop et al. 2024; Sponsler et al. 2020), potentially leading to intense or higher competition during summer. In such systems where midsummer resources are most limiting, pollinators active early in spring are expected to experience somewhat relaxed competition for floral resources because they forage when floral availability may be high (before the summer dearth) yet when relatively few would‐be competitors are active (Sponsler, Hamilton, et al. 2024). At the same time, these early spring‐flying pollinators (e.g., *Celastrina ladon* [spring azure] *Nymphalis antiopa* [mourning cloak], and *Andrena erigeniae* ) may be more limited by abiotic conditions (e.g., low spring temperatures) than by floral resource availability (Herrera 1995; Parker et al. 2016; Stone and Willmer 1989). Insect pollinator species that are active for longer periods of the growing season (e.g., *Bombus* [bumble bee] spp.) may compete with many other species given their wide dietary breath and temporal overlap with flight periods of other insect pollinators (Heinrich 1983). Indeed, *Bombus* spp. are frequently quite flexible temporally in their diets in that they can forage on a wide variety of floral species across the spring and summer depending on spatio‐temporal dynamics of resource availability (Kleijn and Raemakers 2008; Pleasants 1981). Thus, a variety of mechanisms related to insect phenology may drive temporally heterogeneous demand for floral resources, with such demand potentially being strongest in midsummer when floral resources are scarce (Couvillon et al. 2014; Wignall et al. 2020) but thermal conditions most suitable for insect foraging activity (Angilletta Jr et al. 2002; Heinrich 1972; P. G. Willmer 1983).

Several mechanisms may be responsible for variation in floral resource availability over the growing season including species‐specific bloom phenology, climatic factors, pollinator site fidelity, etc. (Kudo and Ida 2013; Ogilvie and Forrest 2017; Phillips et al. 2018). Flower phenology manifests itself in different temporal patterns and degrees of synchronization for various species, sometimes exhibiting extended blooming over long durations or short bursts in an annual cycle (Gentry 1974; P. Willmer 2011). Moreover, floral species vary in their nectar production, with some species (e.g., *Rosa spinosissima* [scotch rose]) producing very little nectar per flower while others produce large volumes (e.g., *Catalpa bignonioides* [southern catalpa]; Southwick et al. 1981). Species‐specific blooming periods coupled with potentially different nectar production patterns could combine to yield variation in nectar availability over the growing season, though very little data exist on nectar availability across a growing season within plant communities (Holl 1995; Potts et al. 2003). Further diversifying resource availability and usage, some bee species exhibit strong floral associations with particular plant species, such as *Bombus griseocollis* (brown‐belted bumble bee) and its strong preference for *Asclepias syriaca* (common milkweed) nectar, which is toxic to most other bee species (Villalona et al. 2020). Clearly, understanding spring and summer floral resource dynamics (i.e., diversity, abundance) is critical to understanding how pollinator communities compete for finite resources across a growing season.

Although questions of resource limitation, use, and variation over time are central to our understanding of how plant‐pollinator communities interact, very little empirical data exist on the temporality of resource availability per se (Baude et al. 2016; Dicks et al. 2012). Instead, many researchers have coarsely estimated floral resource abundance through assessments of flower presence or density (Hegland and Boeke 2006; Hines and Hendrix 2005; Hyjazie and Sargent 2022). However, counts of flowers (or indices of abundance) fail to account for the actual resource sought by many pollinators: nectar and pollen (Sponsler et al. 2023; Szigeti et al. 2016). Furthermore, quantifying resource *presence* is also still not enough to effectively assess competition; instead, direct volumetric measurements of resource availability and subsequent *depletion* (proportion of that available which is used) are the best reasonable approximation of competition intensity (Balfour et al. 2015; Possingham 1988; Sponsler et al. 2023). Moreover, among plant species, depletion can inform pollinator behavior/selection and inform researchers and land managers on which floral species are most sought‐after in a system (Fowler et al. 2016; Possingham 1988). Some of these ideas have recently been tested by Sponsler, Dominik, et al. (2024); Sponsler, Hamilton, et al. (2024) in German orchards and grasslands which have provided a useful framework for considering competition among pollinators.

In the context of nectar depletion over time within communities of plants and pollinators, two mutually exclusive hypotheses have been postulated to explain nectar supply and demand (Sponsler, Hamilton, et al. 2024). The first (equilibrium hypothesis) suggests that competition intensity (as quantified through nectar depletion) should be constant across the growing season because plants and pollinators coevolved and might be expected to optimize the production and use of floral resources like pollen and nectar (Selten and Shmida 1991; Sponsler, Hamilton, et al. 2024). The second (dynamic hypothesis), in contrast, hypothesizes that resource depletion rates may vary across a flowering season, with floral resource “supply” and “demand” being asynchronous through time (Possingham 1988; Ratnieks and Balfour 2021; Sponsler, Hamilton, et al. 2024). Herein, we sampled a complete plant community over the bulk of a growing season in central Kentucky, USA to test whether nectar depletion rates within an early successional old field community more closely adhered to the equilibrium hypothesis or dynamic hypothesis. Given that early successional ecosystems in this region are highly dynamic in terms of plants' physical growth structure and species composition (Askins 2001), we predicted that a dynamic pattern would be observed. We also compared nectar depletion rates among all blooming plant species in the community to assess how species vary in their rewards for insect pollinators.

## 2 Materials and Methods

### 2.1 Study Area and Sampling Design

**FIGURE 1:**
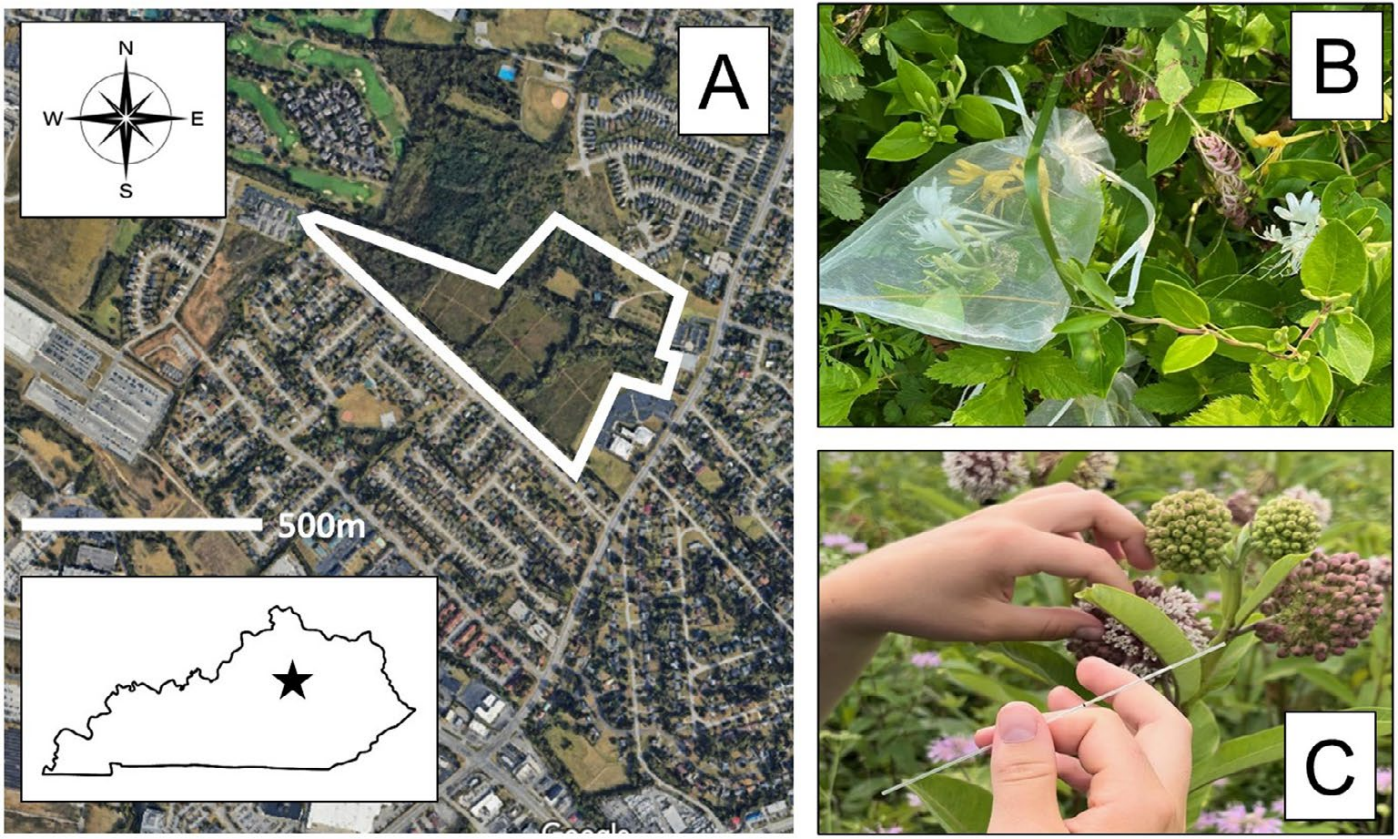
Study area in central Kentucky where we surveyed plant‐pollinator communities (A). The inset map shows where, within Kentucky (black outline) our study area was located (black star, A). Also shown is an aerial image of our study area with the Environmental Research and Education Center (EREC) delineated in white (A). In the study area, we bagged flowers with mesh bags while others were left to be visited by insect pollinators (B). We then sampled nectar from bagged/unbagged flower pairs (C). Photographs by Skylar H. Gillies.

We monitored plant and pollinator communities within the Inner Bluegrass region of Central Kentucky, USA. This area falls within the Interior Plateau ecoregion of the southeastern United States (Drummond 2016). The Inner Bluegrass ecoregion is dominated by a mix of rolling hills and flat plains, being substantially flatter than the Appalachian region to the east but still punctuated by occasional dissected uplands and deeply cut valleys/rivers (Drummond 2016). The soils of this bluegrass section of the interior plateau region are largely alkaline, due to extensive underlying limestone (McNab et al. 2007). Within the Inner Bluegrass region, we studied an early successional old field community at the University of Kentucky's 24‐ha Ecological Research and Education Center (EREC) in Lexington, Kentucky (38.081°N, −84.477°W, WGS84; Figure 1A). This community is kept in a state of early succession via periodic mowing and brush‐hogging to inhibit woody vegetation from advancing in size toward a forested state. The EREC preserve occurs within a strongly suburban context with human development (primarily residential homes and businesses) occurring on all sides (Figure 1A).

Across the EREC preserve, we plotted a 50‐m × 50‐m grid of points in R version 4.2.1 (R Core Team 2023) with the rgdal and raster packages (Bivand et al. 2022; Hijmans 2022). We then masked this grid using a shapefile of the EREC boundary which yielded 103 potential sampling locations. Finally, we assessed each location in the field to ensure feasibility of vegetation sampling (e.g., some points were in standing water or parking lots). The final sample was 90 points, systematically distributed across the EREC. At each of these 90 locations, we marked a transect that was 50 m in length (25 m north and south) and 2 m in width. We surveyed 45 transects each week to conduct flower sampling which gave us a biweekly assessment of floral abundance across the study area. At each 50‐m × 2‐m transect, we conducted a visual count of every flower species and tallied the number of open flowers by species (Mathis et al. 2022). This produced an absolute density of each species at each site, per each visit.

### 2.2 Floral and Nectar Sampling

Since we monitored points on most days of the sampling period, we attempted to extract nectar from all blooming species in the study area. We made attempts to extract nectar from bagged individuals of each species on five occasions on different days. If nectar could never be extracted, it was dismissed from future nectar consideration. For species where nectar was present and could be sampled, we sampled individuals at random locations each day. For each day of nectar sampling, we sampled 1–5 pairs of bagged and unbagged flowers on each focal species. To locate individual plants for bagging, we prioritized bagging individuals on the transect. If no individuals were detectable from the transects sampled that day, we searched in concentric circles for up to an hour for focal species. Bags attached were made of fine mesh that excluded all insect pollinators but did not restrict airflow or sunlight (Sponsler, Dominik, et al. 2024). The bagged individual within each pair was assumed to have the “full” amount of nectar (no depletion) while the un‐bagged individual within each pair was left open for pollinator consumption. Mesh exclosures were deployed in the morning (before 09:00) and collected after approximately 24 h. Nectar was extracted in the field immediately following bag removal using microcapillary tubes (either 0.25 or 5.0 μL). The length of the nectar column was then measured with digital calipers to calculate nectar volume extracted using known volume of the cylindrical microcapillary tubes.

### 2.3 Statistical Analysis

Following Sponsler, Dominik, et al. (2024); Sponsler, Hamilton, et al. (2024), we quantified nectar depletion as the proportional difference between the mean nectar volume of bagged and open flowers. Prior to modeling, we normalized our measurements by dividing each volume reading by the mean volume of the bagged flowers within each species and sampling date. This step served to simplify prior selection (see below) and dampen variation extraneous to the calculation of depletion rate.

To obtain unbiased estimates of mean nectar depletion while respecting the structure of our sampling design, we used a hierarchical Bayesian Gamma regression model with treatment specified as a constant effect and day, species, and plant specified as varying slope and intercept effects (following the nomenclature of Gelman 2005). Proportion of nectar available (i.e., inverse of depletion) was used as the response variable for modeling efficiency. To accommodate the fact that nectar volume measurements are continuously positive but open‐bounded at zero, we included a binomial (i.e., hurdle) submodel with the same specifications as the Gamma model to handle zeros. Priors (Table S1) were selected to be regularizing and weakly informative (Wesner and Pomeranz 2021) following the workflow of Gabry et al. (2019). The model was implemented with Stan (Stan Development Team 2022), accessed via the package *brms* (Bürkner 2017) in R v.4.3.2 (R Core Team 2023). Four chains of 5000 iterations were fit via Hamiltonian Monte Carlo (No U‐Turn Sampler) and included an initial 2500 warmup for each chain, yielding 10,000 posterior samples. We assessed model convergence of parameters via trace plots, Gelman‐Rubin statistics ($\hat{R}$), and bulk and tail effective sample sizes (ESS > 1000; Bürkner 2017). Further, we evaluated model fit using a graphical posterior prediction check to compare empirical and modeled density functions. We used the posterior distributions of nectar availability to calculate the posterior distribution of the mean depletion rate (depletion rate = 1—availability rate).

All other data operations were done in R (R Core Team 2023). Data handling was performed with the tidyverse package suite (Wickham et al. 2019). Model predictions were extracted using tidybayes (Kay 2021b), and models were visualized using *ggplot2* (Wickham 2016), *see* (Lüdecke et al. 2021), and *ggdist* (Kay 2021a). For further description of statistical methods, see Sponsler, Dominik, et al. (2024); Sponsler, Hamilton, et al. (2024).

## 3 Results

**FIGURE 2:**
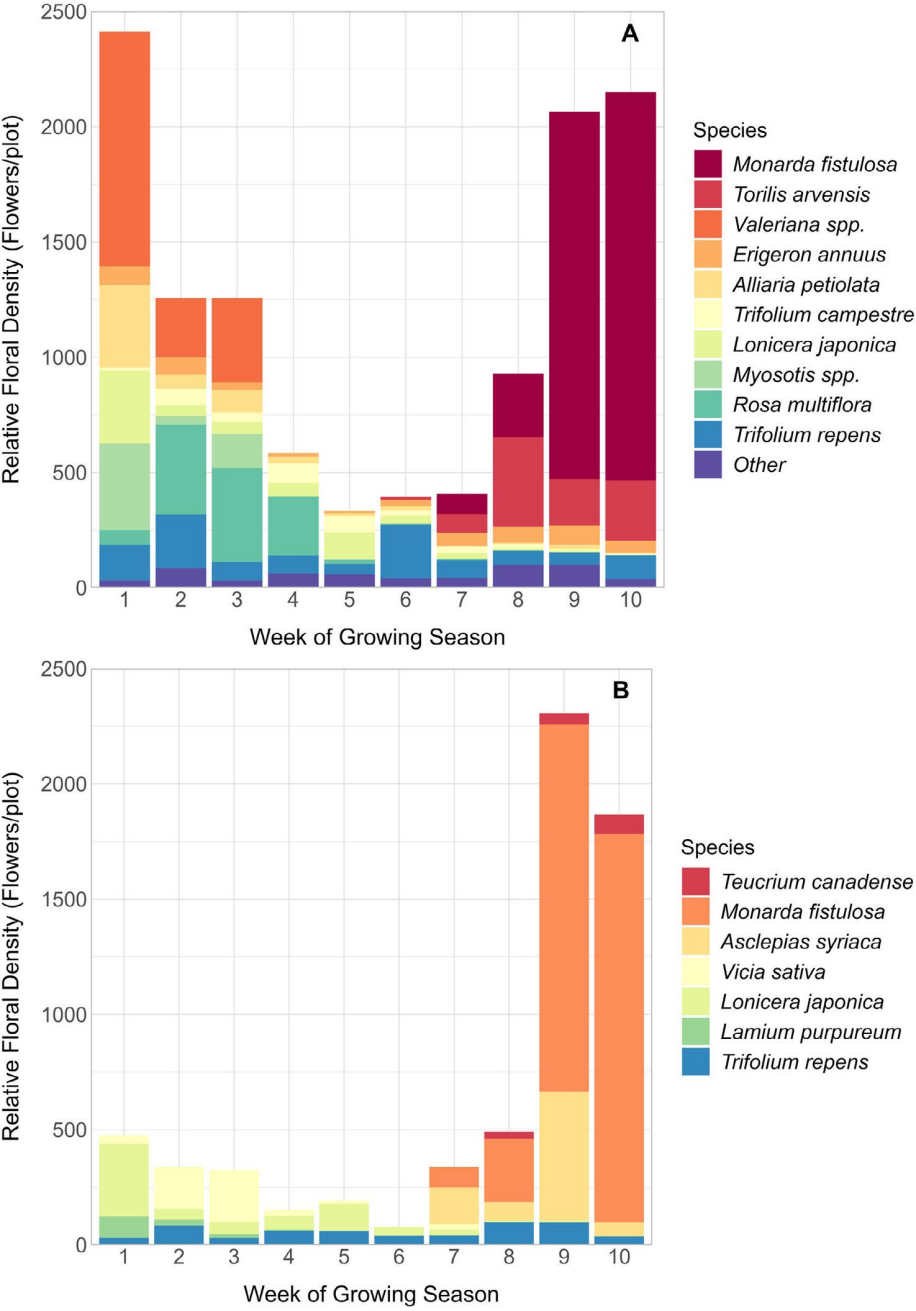
Relative floral density (mean number of flowers per plot) across the 10 weeks of sampling conducted for all flower species observed (A; top 10 most common species delineated with different colors) and this same information presented only for the target species we observed that produced measurable volumes of nectar (B).

From 8 May to 16 July, 2023, we conducted 439 floral density surveys across the EREC preserve. We observed 69 flowering species (67,116 flowers), with *Trifolium repens* (white clover) being the most common (12,307 flowers) and six species each occurring only once (Table S2). Patterns of floral density varied across the 10‐week sampling period (Figure 2A); we observed a bimodal distribution of floral abundance with peaks at week 1 (8–14 May, 2413 flowers per plot) and week 10 (10–16 July, 2150 flowers per plot) and a minimum between weeks 5 and 7 (7–22 June, 335–408 flowers/plot). Among these 69 species, seven produced nectar in measurable quantities: *Teucrium canadense* (American germander), *Monarda fistulosa* (wild bergamot), *Asclepias syriaca* , *Vicia sativa* (common vetch), *Lonicera japonica* (Japanese honeysuckle), *Lamium purpureum* (purple deadnettle), and *Trifolium repens* . Although *Salvia lyrata* (lyreleaf sage) did not fall within any of the established plots, it occurred on the property and was successfully sampled for nectar. When we examined floral density patterns for only these eight nectar‐producing species (Figure 2B), we observed that flower density remained low until the end of the growing season when densities peaked with the bloom of *Monarda fistulosa* (week 9 [3–9 July] = 2307 flowers per plot; week 10 [10–16 July] = 1868 flowers per plot).

During the sampling period, *Lonicera japonica* had the longest bloom duration (present in all 10 weeks of sampling). We only sampled *Teucrium canadense* for 1 week at the end of our field sampling; however, the species was still flowering at the cessation of surveys. We collected 442 bagged/un‐bagged nectar sample pairs with 156 pairs coming from our most‐sampled species, *Trifolium repens* , and 20 pairs coming from our least‐sampled species, *Salvia lyrata* . On average, *Lonicera japonica* produced the most nectar (mean = 2.10 μL per bagged flower) and *Trifolium repens* produced the least nectar (mean = 0.10 μL per bagged flower).

**FIGURE 3:**
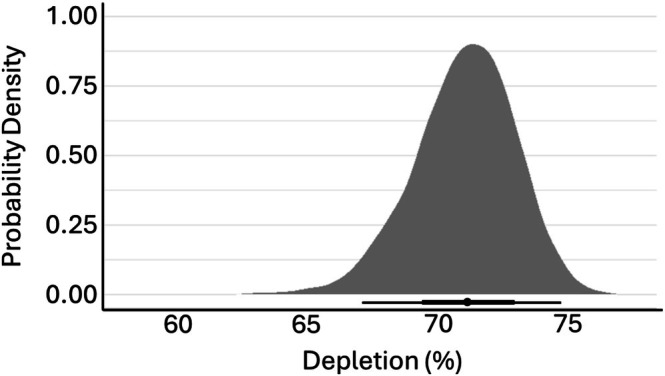
Distribution of nectar depletion rate (%) for flowers visited by pollinators in central Kentucky, 2023. 66% (bold line) and 95% (thin line) credible intervals are included. Depletion rates were calculated using a scaled metric of used vs. available nectar obtained from bagged/unbagged pairs of flowers for each species fit to a Gamma‐Hurdle model. Shown is the average nectar depletion rate across the sampling season (1 May to 15 July) for the average species.

**FIGURE 4:**
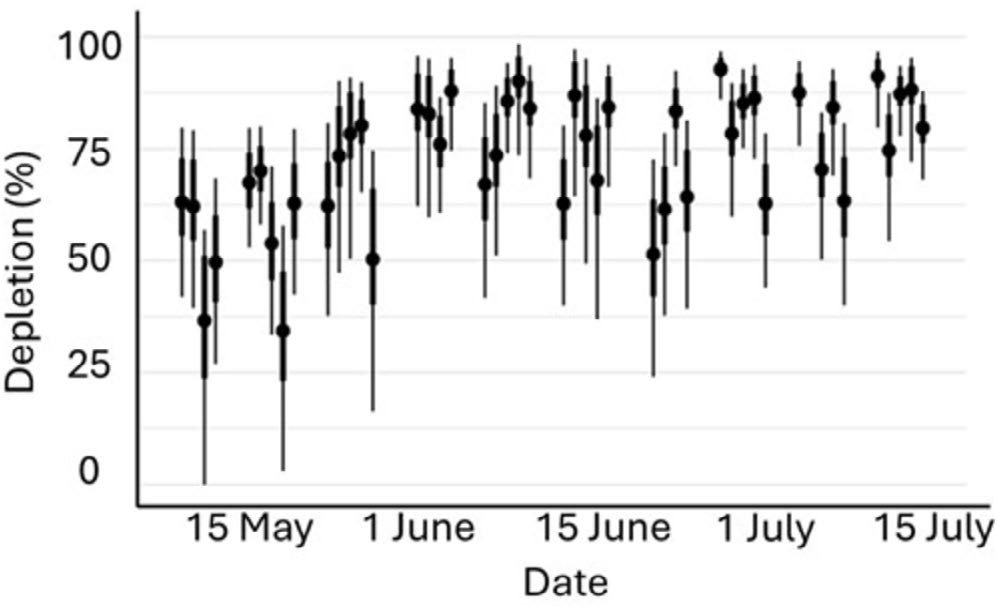
Nectar depletion rates (%) from a floral community in central Kentucky, estimated for each day of the sampling season. 66% (bold line) and 95% (thin line) credible intervals are included. Depletion rates were calculated using a scaled metric of used vs. available nectar obtained from bagged/unbagged pairs of flowers for each species. Estimates were derived from a Gamma‐Hurdle model fit to the nectar depletion data. Shown is the nectar depletion rate across the sampling season (1 May to 15 June) for the average species.

**FIGURE 5:**
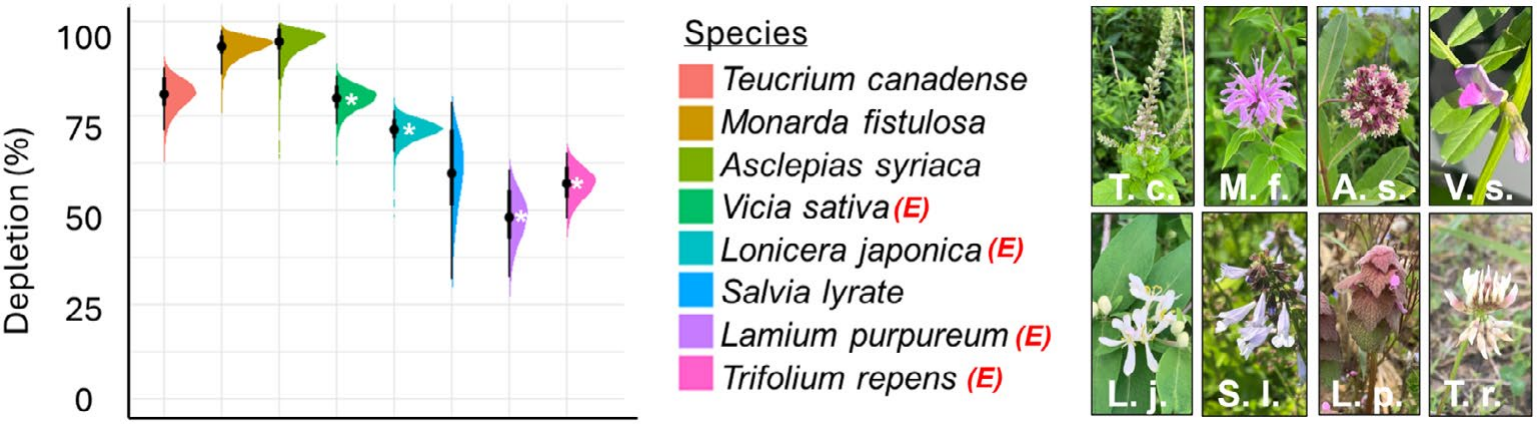
Nectar depletion rates (% available depleted each day; left half of figure) from a floral community in central Kentucky, May–July 2023. 66% (bold line) and 95% (thin line) credible intervals are included. We sampled eight species: *Teucrium canadense (T. c.)*, *Monarda fistulosa (M. f.)*, *Asclepias syriaca (A. s.)*, *Vicia sativa (V. s.)*, *Lonicera japonica (L. j.)*, *Salvia lyrata (S. l.)*, *Lamium purpureum (L. p.)*, and *Trifolium repens (T. r.)*. Images of each of these eight species appear on the right half of the figure. Plotted estimates were derived from a Gamma‐Hurdle model fit to the nectar depletion data. Nectar depletion rates estimated for each species in the model are shown on the plot. Estimates shown with “*” and species names with (E) are exotic to our study area. Photographs by Skylar H. Gillies.

On average, flowers' nectar was depleted at a rate of 71% (95% CI: 67%–75%; Figure 3). Model predictions across the 10‐week survey window indicated somewhat intermediate levels of nectar depletion (e.g., 50%–70%) over the first 3 weeks (9–26 May) which eventually plateaued at a higher level (80%–85%) as the growing season advanced (30 May–14 July; Figure 4). Among focal species, we observed considerable variation in nectar depletion rates (Figure 5). Specifically, *Lamium purpureum* was the least depleted (on average, 48% of its nectar depleted, 95% CI: 32%–61%), followed by *Trifolium repens* (57% depleted, 95% CI: 48%–65%). In contrast, *Asclepias syriaca* was the most depleted (on average, 95% depleted, 95% CI: 85%–99%), followed by *Monarda fistulosa* (93% depleted, 95% CI: 86%–98%; Figure 5).

**FIGURE 6:**
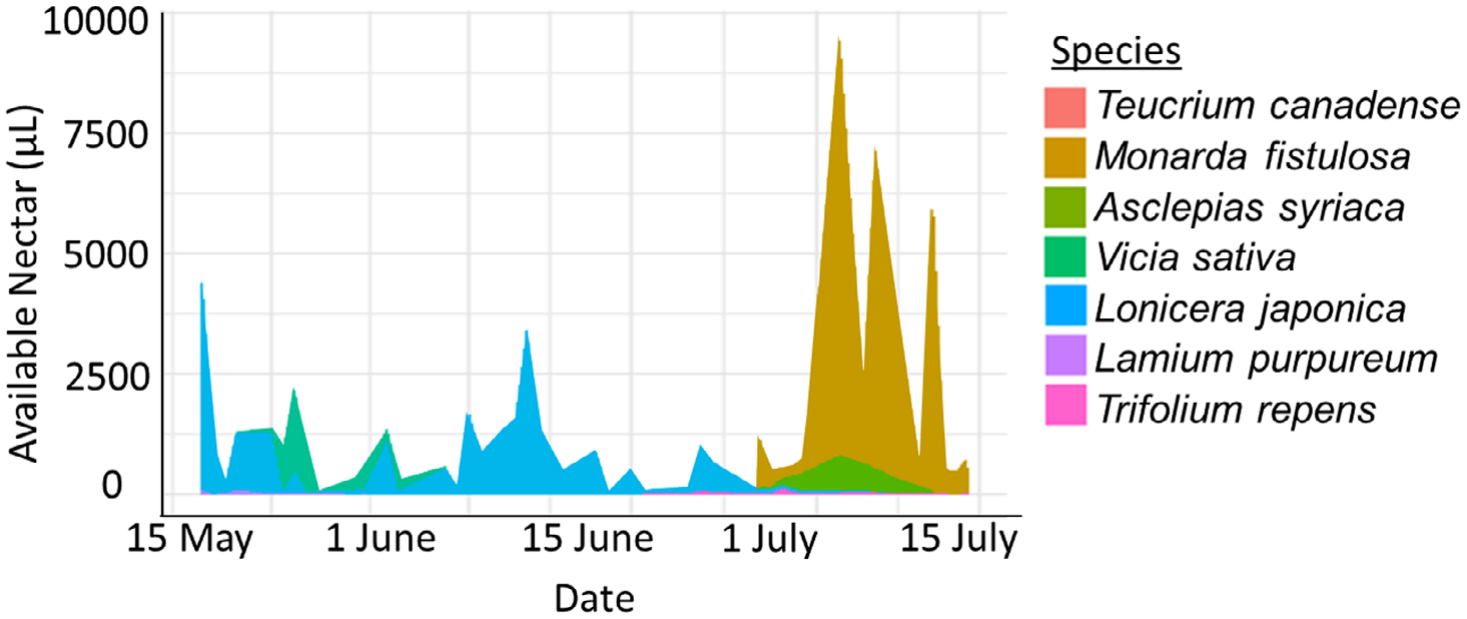
Available nectar (μL) within floral communities from eight species of nectar‐producing plants in central Kentucky, May–July 2023. Available nectar was estimated using nectar crops sampled from bagged plants in situ using microcapillary tubes fit with a Gamma‐Hurdle model and flower counts from across the study area. Although we extracted nectar from *Salvia lyrata* , this species was present at low densities and was not observed in any sample plots. Thus, *Salvia lyrata* is not present in this figure.

After correcting for species‐specific abundance, *Vicia sativa* and *Lonicera japonica* produced the greatest quantity of nectar over the first 7 weeks, after which time *Asclepias syriaca* and *Monarda fistulosa* , which were both abundant and heavy nectar producers, yielded a 2–3× increase in available nectar for pollinators (Figure 6).

## 4 Discussion

Although a body of theoretical literature suggests that the equilibrium hypothesis (constant rates of nectar depletion across a growing season) may be supported in certain systems and circumstances, our data adds to a growing body of evidence suggesting that the dynamic hypothesis (variable rates of depletion across a growing season) may be common in a variety of temperate plant‐pollinator communities (Possingham 1988; Ratnieks and Balfour 2021; Sponsler, Hamilton, et al. 2024). The dynamic nectar depletion pattern has now been observed on two continents and three ecological contexts: this study (North America, shrubland community) and (Sponsler, Dominik, et al. (2024) and Sponsler, Hamilton, et al. (2024), Europe, grasslands and agricultural lands). With that in mind, all three of these studies to date have been conducted within highly dynamic ecological communities (i.e., those dependent upon disturbance or characterized by shifting plant communities; Askins 2001), suggesting the need for additional work quantifying nectar dynamics within more stable ecological communities. Beyond our support for the dynamic hypothesis, our finding that nectar depletion rates climbed to a peak of ~80% and remained high may be explained by several ecological phenomena. The low rates of depletion early in the growing season might be driven by relatively low pollinator abundance (e.g., *Bombus* spp. are at their lowest densities; Aizen 2001; Rollings and Goulson 2019), poor thermal conditions (e.g., rain, cold, excessive heat; Willmer 1983), or high exotic floral abundance—especially *Lonicera japonica* . Later in the growing season, central Kentucky is warmer and drier (Hill 2005; Tekoe and Suriano 2025), which is often suitable for ectotherm foraging (Deal 1941; Peat and Goulson 2005; Willmer and Stone 2004). Finally, both *Bombus* spp. (especially *B. impatiens* [common eastern bumble bee]) and *Apis mellifera* , both of which are abundant at our study site, are at peak density in the mid‐late growing season (Knoll et al. 2020; Timberlake et al. 2020; Treanore et al. 2020), potentially amplifying competition.

A key observation from our study was that pollinators left up to 1/3 of available nectar, on average, unused within the study system (Figure 3). Incomplete use of available nectar raises an interesting question: Why did pollinators leave so much nectar “on the table”? Such a pattern could arise via several means. Perhaps, insect‐pollinated plants in this system produce excessive amounts of nectar than is required to attract their needed pollinators as means for outcompeting nearby plants (Ratnieks and Balfour 2021). Alternatively, insect pollinators may be somewhat inefficient at extracting nectar from the landscape and the ~70% depletion rate we observed is the effective “maximum” depletion rate that can reasonably be achieved (Pleasants 1989; Pyke 1984). A third explanation is that not all flowers/nectar are of equal value to the pollinator community we observed (Fowler et al. 2016; Rollings and Goulson 2019). This might be the case for specialized flowers that exclude generalist pollinators or when nectar quality (e.g., sugar concentration) varies among species (Southwick et al. 1981). As discussed below, our findings support the idea that different plant species vary in floral rewards as, at least, a contributor to incomplete nectar use within this community. From a conservation perspective, interpretation of incomplete nectar depletion remains somewhat ambiguous; for example, if nectar crops are totally depleted, this could be viewed in a positive light as pollinators have benefited to the greatest extent possible from a given species' nectar stores (Cnaani et al. 2006; Harris et al. 2024). Conversely, if nectar stores are depleted, this resource may be limiting pollinator populations and may require enhancement through the application of conservation action (Harris et al. 2023; Ratnieks and Balfour 2021). This latter line of thought might indicate that unused nectar stores are a positive because they represent available nectar for pollinators (Pleasants 1981). Finally, studies such as that presented here occur against a backdrop of widespread insect pollinator declines (Goulson et al. 2015; Potts et al. 2010). Clearly, in communities where pollinator community health is highly degraded, nectar depletion rates may be artificially deflated. Future work examining nectar depletion dynamics within habitats of varying quality would be valuable in helping to inform this topic.

Beyond community‐wide nectar depletion rates, our results point to strong heterogeneity in pollinator depletion rates of nectar‐producing plant species (Figure 5). This is interesting because, if all flowering plants in the system were functionally the same, they would be depleted at the same rates (Heinrich 1975). Our observations contrasted strongly with this idea whereby species like *Monarda fistulosa* were consistently drained of its nectar while *Trifolium repens* was left with nearly half its nectar unused (Figure 5). Although future work is needed to understand the drivers behind this heterogeneity, some potential drivers may be differences in nectar quality (Southwick et al. 1981), variation in pollen availability and quality (Harder 1990), or non‐optimal foraging behavior by pollinators (Heinrich 1983). Additionally, heterogeneous use of plant species may be influenced by uneven availability of flowers across space (Williams and Kremen 2007) and, for most species, time (Russo et al. 2013; Timberlake et al. 2020). A final point of consideration is that variable depletion rates seem to indicate variable value to pollinators, with depleted species being the most valuable to pollinating insects (Figure 5). In particular, natives *Asclepias syriaca* and *Monarda fistulosa* were two of the most depleted species and the former, in particular, produced the most nectar per flower of any species we sampled. Thus, conservationists aiming to support the availability of nectar in central Kentucky should consider the inclusion of these two species when designing pollinator plantings (Baker and Potter 2018; Cruden et al. 1984).

Although species‐specific depletion rates provide an important view into the pollination dynamics within our system, the addition of floral abundance data allowed us to scale our raw nectar data to obtain an estimate of total relative volume of each species across the study area (Figure 6). Peak nectar volume largely coincided with peak nectar depletion rates (i.e., early July) and was driven largely by *Monarda fistulosa* . Although *Asclepias syriaca* was also blooming during this time and produced a large volume of nectar (Figure 5), the high abundance of *M. fistulosa* meant that this species generated a much greater volume of nectar available for pollinators. Although *M. fistulosa* is native to Kentucky (Barnes 1998), another species that produced large volumes of nectar in our study system, *Lonicera japonica* , is an invasive exotic (Schierenbeck 2004). The apparent value of *L. japonica* in providing nectar to pollinators through May, June, and early July is problematic because this widespread invasive causes immense ecological damage to native ecosystems across Kentucky (Liang 2010; Liu et al. 2011). Although we do not advocate for the promotion of *L. japonica* as a food source for pollinators in Kentucky, it is worth noting that its presence does provide nectar resources for insect pollinators (Seitz et al. 2020; Valdovinos et al. 2009).

Our paper provides strong support for the dynamic hypothesis of nectar depletion within a temperate, early successional plant community (Possingham 1988; Ratnieks and Balfour 2021; Sponsler, Hamilton, et al. 2024) and demonstrates variation in nectar depletion by species. However, many aspects of this topic remain completely unanswered. For instance, we predicted that the dynamic hypothesis would best explain nectar depletion patterns within this community because early successional woody communities host high species turnover (Wright and Fridley 2010), however, the high abundance of exotic species (42% of species and 70% of counted flowers; Table S2) complicates expectations of how such systems may behave. Abundant exotic species may contribute a great degree of ecological “noise” into the system from a nectar perspective because they evolved within a different region and, perhaps, in equilibrium within a different plant community (Selten and Shmida 1991). However, given that abundant exotic species is largely the norm across many plant communities across North America (Fan et al. 2013), our findings may reflect a fairly typical pattern present in many contemporary plant‐pollinator networks across much of the continent. Monitoring intact, native pollinator ecosystems, perhaps across a spectrum of conditions (e.g., age classes and ecological stability) would be valuable (Cariveau et al. 2020; Parrish and Bazzaz 1979). Beyond the ecological context of our study, we did not examine pollen availability, which overlooks a key resource required by many insect pollinators (e.g., most bees; Jones 2012). Quantifying pollen availability, in addition to nectar, would clearly provide a more complete picture of the floral resources available to pollinators (Hicks et al. 2016). Indeed, several species we sampled for nectar (e.g., *Viola sororia* [common blue violet]) never yielded measurable quantities of nectar, despite many attempts to locate it, perhaps because such species exhibit high rates of cleistogamy (Solbrig et al. 1980). Pollinators visiting plant species lacking nectar, perhaps, focus their foraging efforts on pollen resources (Palmer‐Young et al. 2018). We also did not quantify nectar quality. From a mechanistic perspective, data on pollinator abundance (perhaps using detection‐adjusted methods such as those described by McNeil et al. 2019) would also help inform the drivers behind observed nectar depletion rates reported here. Future work that quantifies differences in resource concentration within nectars produced by different species would be valuable (*sensu* Southwick et al. 1981). Regardless, it is our hope that by conducting the first analysis of nectar depletion across a broad plant community in North America, we demonstrate the value of these data for understanding plant‐insect interactions.

## Author Contributions

**Skylar H. Gillies:** conceptualization (equal), data curation (equal), formal analysis (equal), investigation (equal), methodology (equal), validation (equal), visualization (equal), writing – original draft (equal), writing – review and editing (equal). **Douglas B. Sponsler:** conceptualization (equal), data curation (equal), formal analysis (equal), methodology (equal), validation (equal), visualization (equal), writing – review and editing (equal). **Zachary J. Hackworth:** conceptualization (equal), data curation (equal), formal analysis (equal), methodology (equal), validation (equal), visualization (equal), writing – review and editing (equal). **Darin J. McNeil:** conceptualization (equal), funding acquisition (equal), investigation (equal), methodology (equal), project administration (equal), resources (equal), supervision (equal), validation (equal), writing – original draft (equal), writing – review and editing (equal).

## Funding

This work was supported by United States Department of Agriculture ‐ National Institute of Food and Agriculture McIntire‐Stennis Capacity Grant, #KY009043 and a University of Kentucky ‐ Department of Biology Funkhouser Summer Research Internship.

## Conflicts of Interest

The authors declare no conflicts of interest.

## Supporting information


**Table S1:** Prior distributions for the gamma and binomial components of the gamma‐hurdle model used to predict nectar depletion.
**Table S2:** All flower species observed on transects for this study. For each species, we also present the family name, status (native [N] or exotic [E]), count type (flowers [F] or inflorescences [I]), number counted during surveys (count), and the number of paired (bagged/unbagged) nectar samples obtained (Nectar samples).

## Data Availability

The data presented here is archived on github and can be accessed using the following link: https://github.com/darinjmcneil/nectar. The data will also be available on dryad with the following dataset DOI: 10.5061/dryad.jm63xsjr4.
