# Supplementary material for: Good to the Last Drop? Nectar Depletion by Insect Pollinators Varies Among Plant Species and Through Time
Source: Ecol Evol. 2026 Jul 14;16(7):e74008. doi: 10.1002/ece3.74008 (PMC13368463; doi:10.1002/ece3.74008)
Supplement: Supplementary file 1 — Table S1: Prior distributions for the gamma and binomial components of the gamma‐hurdle model used to predict nectar depletion. Table S2: All flower species observed on transects for this study. For each species, we also present the family name, status (native [N] or exotic [E]), count type (flowers [F] or inflorescences [I]), number counted during surveys (count), and the number of paired (bagged/unbagged) nectar samples obtained (Nectar samples). [file ECE3-16-e74008-s001.docx]

**Table S1.** Prior distributions for the gamma and binomial components of the gamma hurdle model used to predict nectar depletion.

| Model Component | Parameter Class | Prior |
| --- | --- | --- |
| Gamma | Intercept | N(0, 0.1) |
|  | Treatment Coef | N(-1.5, 1) |
|  | SD | N(0, 1) |
|  | Shape | N(0, 5) |
| Binomial | Intercept | N(-1, 1.5) |
|  | Treatment Coef | N(1.5, 1.25) |
|  | SD | N(0, 2) |

**Table S2.** All flower species observed on transects for this study. For each species, we also present the family name, status (native [N] or exotic [E]), count type (flowers [F] or inflorescences [I]), number counted during surveys (count), and the number of paired (bagged/unbagged) nectar samples obtained (Nectar samples).

| **Family** | **Scientific name** | **Status** | **Count type** | **Count** | **Nectar samples** |
| --- | --- | --- | --- | --- | --- |
| Acanthaceae | *Ruellia caroliniensis* | N | F | 1 | - |
| Amaryllidaceae | *Allium vineale* | E | F | 27 | - |
| Apiaceae | *Conium maculatum* | E | F | 143 | - |
|  | *Pastinaca sativa* | E | I | 33 | - |
|  | *Torilis arvensis* | E | F | 3,363 | - |
| Apocynaceae | *Apocynum cannabinum* | N | F | 72 | - |
|  | *Asclepias syriaca* | N | F | 30 | 30 |
|  | *Asclepias tuberosa* | N | F | 14 | - |
| Asteraceae | *Achillea millefolium* | N | F | 191 | - |
|  | *Carduus nutans* | E | I | 1 | - |
|  | *Cichorium intybus* | E | F | 3 | - |
|  | *Echinacea purpurea* | N | I | 3 | - |
|  | *Erigeron annuus* | N | I | 2,215 | - |
|  | *Hieracium caespitosum* | E | I | 1 | - |
|  | *Jacobaea vulgaris* | E | I | 168 | - |
|  | *Rudbeckia hirta* | N | I | 39 | - |
|  | *Sericocarpus sp.* | N | I | 10 | - |
|  | *Taraxacum officinale* | E | I | 118 | - |
|  | *Vernonia gigantea* | N | I | 7 | - |
| Balsaminaceae | *Impatiens capensis* | N | F | 1 | - |
| Boraginaceae | *Myosotis macrosperma* | N | F | 586 | - |
| Brassicaceae | *Alliaria petiolata* | E | F | 1,388 | - |
|  | *Barbarea vulgaris* | E | F | 34 | - |
|  | *Capsella bursa-pastoris* | E | F | 295 | - |
|  | *Lepidium virginicum* | N | F | 14 | - |
| Caprifoliaceae | *Dipsacus fullonum* | E | F | 8 | - |
|  | *Lonicera japonica* | E | F | 4,658 | 122 |
|  | *Valerianella locusta* | E | F | 2,741 | - |
| Caryophyllaceae | *Arenaria serpyllifolia* | E | F | 330 | - |
|  | *Cerastium sp.* | N | F | 1,533 | - |
|  | *Dianthus armeria* | E | F | 33 | - |
| Commelinaceae | *Commelina communis* | E | F | 3 | - |
| Convolvulaceae | *Calystegia sepium* | N | F | 1 | - |
|  | *Convolvulus arvensis* | E | F | 6 | - |
| Cornaceae | *Cornus racemosa* | N | F | 8 | - |
| Fabaceae | *Robinia pseudoacacia* | N | F | 17 | - |
|  | *Trifolium campestre* | E | I | 7010 | - |
|  | *Trifolium pratense* | E | I | 8 | - |
|  | *Trifolium repens* | E | I | 12370 | 39 |
|  | *Vicia sativa* | E | F | 2826 | 42 |
| Geraniaceae | *Geranium maculatum* | N | F | 1216 | - |
| Hydrophyllaceae | *Phacelia purshii* | N | F | 40 | - |
| Hypericaceae | *Hypericum punctatum* | N | F | 15 | - |
| Iridaceae | *Sisyrinchium sp.* | N | F | 1 | - |
| Lamiaceae | *Glechoma hederacea* | E | F | 138 | - |
|  | *Lamium purpureum* | E | F | 1,209 | 36 |
|  | *Monarda fistulosa* | N | F | 7,512 | 29 |
|  | *Prunella vulgari* | N | F | 10 | - |
|  | *Pycnanthemum sp.* | N | F | 268 | - |
|  | *Teucrium canadense* | N | F | 47 | 7 |
| Oleaceae | *Ligustrum obtusifolium* | E | F | 2 | - |
| Oxalidaceae | *Oxalis dillenii* | N | F | 24 | - |
|  | *Oxalis sp.* | N | F | 842 | - |
| Phytolaccaceae | *Phytolacca americana* | N | F | 12 | - |
| Plantaginaceae | *Plantago lanceolata* | E | I | 141 | - |
|  | *Veronica sp.* | N | F | 3,797 | - |
| Ranunculaceae | *Ranunculus fascicularis* | N | F | 10 | - |
|  | *Ranunculus sp.* | N | F | 31 | - |
| Rosaceae | *Geum canadense* | N | F | 176 | - |
|  | *Potentilla sp.* | N | F | 283 | - |
|  | *Rosa multiflora* | E | F | 10,281 | - |
|  | *Rosa setigera* | N | F | 15 | - |
| Rubiaceae | *Galium sp.* | N | F | 128 | - |
|  | *Sherardia sp.* | N | F | 567 | - |
| Solanaceae | *Solanum carolinense* | N | F | 60 | - |
| Verbenaceae | *Verbena hastata* | N | F | 21 | - |
| Violaceae | *Viola arvensis* | E | F | 3 | - |
|  | *Viola sororia* | N | F | 8 | - |
